# His Hormone, Her Oogenesis: How Male Malaria Mosquitoes Trigger Female Egg Development

**DOI:** 10.1371/journal.pbio.1001694

**Published:** 2013-10-29

**Authors:** Richard Robinson

**Affiliations:** Freelance Science Writer, Sherborn, Massachusetts, United States of America

A female mosquito needs two things to reproduce: your blood and a visit from a male mosquito. The blood provides the nutrient-rich meal she will use to feed her developing eggs. The male provides not only sperm, but also a complex mix of proteins and other molecules, secreted from the so-called male accessory glands (MAGs) and deposited along with the sperm during mating. The secretions form a gelatinous “mating plug,” which blocks access to the uterus.

**Figure:**
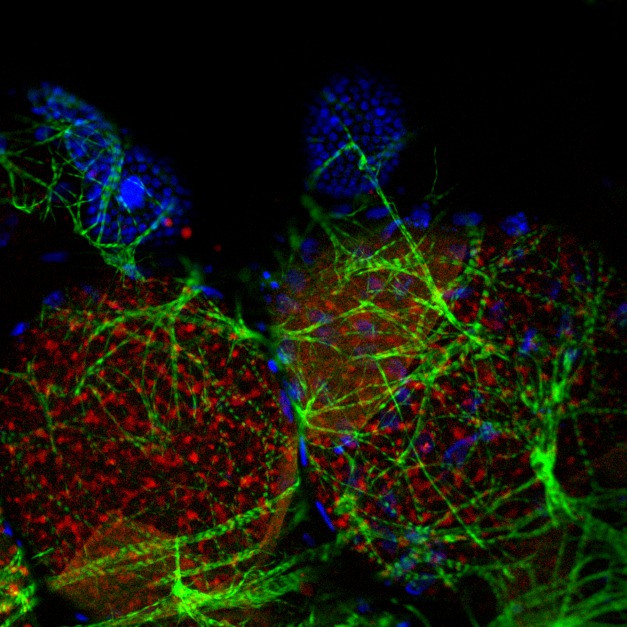
*Anopheles* oocytes after blood feeding. The oocyte, which is surrounded by epithelial cells (blue) and muscle cells (green), accumulates lipids (red) in the cytoplasm. This accumulation depends on the interaction between a female protein, MISO, and the male hormone 20E.

Both the blood meal and copulation provide necessary signals for the female to begin development of eggs in her ovary. While much is known about the events triggered by feeding, relatively little is known about how mating stimulates oogenesis. A new study by Francesco Baldini, Flaminia Catteruccia, and colleagues shows that a hormone delivered by males stimulates production of a protein in females, and together the pair induces expression of multiple genes to increase egg development.

Working with the malaria-carrying mosquito *Anopheles gambiae*, the authors had previously shown that a protein called mating-induced stimulator of oogenesis (MISO) was upregulated in the uterus, or atrium, shortly after mating. They began the current work by using RNA interference to block MISO expression in mated, blood-fed females. They found that oogenesis specifically was impaired, decreasing egg development in treated insects to the level found in virgin females. Those egg follicles that did develop did so slowly, suggesting that they were unable to accumulate lipids at the normal rate. This, the authors found, was due to decreased expression of the lipid transporter lipophorin, whose levels were again similar to those seen in virgin females, as a consequence of loss of MISO.

Previous studies have suggested that MAG secretions control aspects of behavior and physiology in females after mating and that one key component of the secretions is the steroid hormone 20-hydroxy-ecdysone (20E), which has multiple effects in female mosquitoes. Control and MISO-deficient females had equivalent levels of 20E in the mating plug, but it dispersed from the plug much more slowly in the females with reduced MISO. This suggested that the two might interact, a hypothesis confirmed when the authors showed that 20E and MISO co-immunoprecipitated. Loss of circulating 20E led to reduced expression of several of its gene targets, including the yolk protein precursor vitellogeninand the ecdysone receptor (EcR), which acts as nuclear receptor for the hormone. In control mosquitoes, an increase in 20E led to an increase in production of MISO, an effect that could be blocked by loss of its receptor EcR. The picture that emerges is that male-supplied 20E binds to EcR to induce the female to produce MISO, which it then binds as well. This complex then promotes transcription of a variety of oogenic genes, leading to increased lipid accumulation in oocytes and promoting egg development.

The elucidation of this pathway may have important consequences in the fight against malaria, which yearly kills over half a million people worldwide. Introduction of males deficient in 20E synthesis, for instance, could reduce reproduction in females that mate with them. Furthermore, the lipid accumulation that occurs during oogenesis reduces the ability of the female mosquitos to destroy the malaria parasites they carry; thus, blocking this pathway could reduce the deadliness of a bite from these troublesome insects.

**Baldini F, Gabrieli P, South A, Valim C, Mancini F, et al. (2013) The Interaction between a Sexually Transferred Steroid Hormone and a Female Protein Regulates Oogenesis in the Malaria Mosquito** ***Anopheles gambiae*** **. doi:10.1371/journal.pbio.1001695**

